# How is a psychotherapeutic process like a psychedelic drug? Neurocognitive evidence for a novel mechanism of action with Regenerating Images in Memory

**DOI:** 10.3389/fpsyg.2025.1539079

**Published:** 2025-05-15

**Authors:** Paul F. Cook, Laurra M. Aagaard, Lisa Krug Avery, Nichole Long, Allen Alford, Deborah Sandella

**Affiliations:** ^1^University of Colorado College of Nursing, Aurora, CO, United States; ^2^RIM Institute, Denver, CO, United States

**Keywords:** brief therapy, cognitive neuroscience, EEG, imagery, mystical experience, stress, trauma

## Abstract

**Objective:**

Nursing students are at risk for traumatic stress, but current treatments have limited benefits. Regenerating Images in Memory (RIM) is a verbal psychotherapeutic approach to help people safely re-experience troubling memories, then integrate them into meaningful life narratives. RIM’s developers propose a mechanism of action based on nonconscious processes like emotional processing and body awareness. Two Minds Theory (TMT) is a health behavior model that suggests coping arises primarily from the speedy and non-conscious Intuitive Mind. This study was designed to test a potential mechanism of action for RIM based on TMT, where the Intuitive Mind suggests solutions that are later integrated into the Narrative Mind.

**Methods:**

In this exploratory, descriptive, mechanistic study with no comparison group, 30 nursing students received RIM during the late COVID-19 pandemic. Participants completed validated symptom questionnaires before and after 1 RIM session lasting 1–2 h. As an exploratory measure, we measured altered states of consciousness using a scale linked to rapid improvement in studies of psychedelic treatments. Sessions were audio-recorded and participants’ brainwaves were monitored using a MUSE S (Gen 1) 5-lead EEG headband with MindMonitor software.

**Results:**

Students reported pre-post improvements (Cohen’s *d* = 1.93–2.75) on 4 of 5 questionnaires. Participants reported levels of altered consciousness similar to those in psychedelic drug studies, particularly on an “ineffability” subscale linked to symptom improvement. EEG readings showed a significant shift away from the frontal lobes (associated with the Narrative Mind) and into the temporal lobes (associated with the Intuitive Mind), *χ^2^* = 11.0 × 10^4^, *p* < 0.001, during the middle stage of RIM. This was followed by frontal and temporal lobe co-activation during the final stage of RIM, a finding that also mirrors psychedelic studies’ finding of increased synchronization across brain areas during treatment.

**Conclusion:**

Replicating prior pre-post RIM studies, nursing students reported symptom improvement. These changes co-occurred with altered consciousness and increased temporal-lobe brain activity, findings that are consistent with RIM’s proposed mechanism of action based on TMT. Although this uncontrolled trial does not allow conclusions about treatment efficacy, RIM is a brief verbal psychotherapeutic intervention that merits further study.

## 1 Introduction

Stress is a common human experience, but both the prevalence and severity of stress reactions rose substantially in the general U.S. population since 2020 (American Psychological Association, 2024; Jia et al., 2021). Across the world the prevalence of more serious traumatic stress reactions including post traumatic stress disorder (PTSD) also continues to rise (Oakley et al., 2021). People in certain professions have greater exposure to trauma, and therefore greater risk for traumatic stress (Schein et al., 2021). Nursing students are a group at especially high risk for traumatic stress reactions in recent years because of pre-existing risk factors, differential stressors created by the COVID-19 pandemic, and nursing workforce shortages. Excess occupational stress predisposes nurses to stress injuries and burnout (Sampaio et al., 2021). Many universities therefore launched programs to assess and improve student mental health in the wake of the COVID-19 pandemic (e.g., Peterson et al., 2023).

Despite the recognized importance of mental health and the need for effective interventions, contemporary treatments can have disappointing results. Medications have relatively weak effects for PTSD, with a standardized effect size of *d* = 0.43 for paroxetine and smaller effects for other commonly used antidepressants (Guidetti et al., 2024). Cognitive-behavioral therapy (CBT) is the leading psychotherapeutic approach for PTSD, with average effects around *d* = 0.75 (Cusack et al., 2016) that may still leave patients with significant symptoms. Although this is slightly better than medication, CBT has similar problems with attrition due to stigma and the amount of time and effort required for treatment (Cusack et al., 2016; Kolovos et al., 2016). Eye movement desensitization and reprocessing (EMDR) is widely used for PTSD, although it is supported by less evidence than CBT (American Psychological Association, 2017); multiple reviews suggest that its effects are either equivalent to CBT (Perlini et al., 2020) or at best slightly superior (Chen et al., 2015). Aerobic exercise is also effective for PTSD symptoms (Björkman and Ekblom, 2022), but has its own problems with dropout and can be unappealing to patients who are also depressed (Kvam et al., 2016). Due to the limited existing treatment options, mental health researchers are exploring a wide range of new modalities for PTSD such as ketamine (Feder et al., 2020), transcranial magnetic stimulation (TENS: Feng et al., 2019), and psychedelic drugs (Artru and Rabeyron, 2021; Henner et al., 2022). These novel treatments promise rapid symptom relief, but may carry unknown risks.

### 1.1 Regenerating Images in Memory

Regenerating Images in Memory (RIM) is a novel verbal psychotherapeutic approach for PTSD (Sandella, 2016). RIM’s underlying causal mechanism may link to the finding that traumatic memories are stored differently than other memories, affecting people’s physical experiences and reactions without becoming part of the ongoing narrative of their lives (Van der Kolk, 2014). In particular, negative emotional memories tend to be experienced in a more analytic, data-driven fashion that is isolated from emotions, while positive memories activate areas across the entire brain (Levine and Pizarro, 2004). In treatment, it is frequently the case that simply talking about a traumatic memory does not “unlock” it from the analytic mode of processing, so the patient’s symptoms do not change (Nader et al., 2000). RIM is hypothesized to allow for new experiential learning by first (A) inviting people to turn their focus from the external world to an inner awareness of body sensation and spontaneously flowing imagery, in a client-generated process that promotes a sense of control, creates an unconscious sense of safety, and allows initial memories of terror and helplessness to rise into awareness. RIM skills are then used to (B) quickly guide the client to regenerate these memories into an empowered version that is saved in memory as an experience of safety, with freedom to speak and move at will. A sense of bodily awareness is essential for this new experience, based on fMRI evidence that imagining oneself in an activity generates brain patterns that are similar to actually engaging in it, and different from those associated with talking about the activity (Taube et al., 2015). Finally, (C) fragmented past memories are integrated into new and meaningful life narratives, which are then more accessible (Nader, 2015) and able to promote more adaptive behavior in everyday life.

The 3-phase process within a single RIM session (steps A through C above) is described as “*dip*-*see*-*do*” (Sandella, 2016). In the initial *dip* phase, the client usually has their eyes closed and begins with slow breathing; the RIM facilitator brings the client’s attention first to their breath, and then to other areas of their body. By encouraging rich description of the client’s embodied experience, the RIM facilitator helps the client to experience and verbalize imagery (color, texture, shape, size) rather than detached observations or descriptions. This imagery then naturally resolves into a core traumatic memory or conflict, which might be internal or might involve other people. The RIM facilitator explores the client’s awareness of that memory, encouraging a more empowered and active stance but not instructing the client in deliberate coping strategies. Once the memory leads to an emotional resolution, the third phase involves helping the client return their awareness to the outside world and to envision future activities carried out in the context of that success. Observation of RIM sessions suggests that the most intense imagery-based experiences do indeed occur during the middle *see* phase, and that these experiences are often surprising in their level of intensity and personal meaning (Cook, in press).

The following recent clinical example illustrates the process of a RIM intervention:

A woman whose daughter was present and lost her best friend during a high school shooting requested a RIM session about 1 year later. She felt unable to settle her body and had restless legs, despite running 10 miles per week to cope. Her daughter had requested some time apart because the mother’s hypervigilance was “driving her crazy.” As the mother sat with her eyes closed and tuned in to her body during RIM, she sensed a ball of string in her stomach. Guided to zoom in closer to the ball, she said with surprise, “it’s a ball of worms and they are eating my stomach from the inside.” At this moment, she was asked to have her imagination bring forth an image of the source of this feeling. Instantly, she felt herself spontaneously transported to her car, parked outside of the locked-down school where her daughter was detained. As the mother began giving words to her feelings, she said “I just want to run inside, grab her, and bring her here with me.” The facilitator said, “OK, do it.” The mother quickly said, “I’m running into the classroom where it all happened. Now I understand what my daughter has been describing. … I find her [the daughter] and grab her, and we are running back to the car. Now we are sitting in the front seat hugging each other, and I’m holding her as tight as I can, and it feels so good. I have not felt safe until now.” She was encouraged to continue the hug for as long as she needed, and when it felt complete, her restlessness was also gone and a feeling of safety and connection was present again. After that experience, the mother’s hovering behavior disappeared, and her relationship with her daughter again became playful and positive, as they planned and shopped for the daughter’s first year of college.

Although not yet supported by a high level of evidence, RIM is currently available in the community, and has shown beneficial effects in preliminary studies. These include pre-post pilot studies of patients with varied histories of trauma (Ellis Ecke, 2018; Kneier, 2016; McDonald, 2017; Prah, 2015; Waidtl, 2015), a non-randomized trial to improve quality of life among patients with irritable bowel syndrome (*n* = 38 with group assignment by self-selection: Boxwell and Eichler, 2006), and a pilot randomized trial of RIM versus group coaching for unresolved grief (*n* = 12: Elle and Alberts, 2023). The method therefore is considered promising, but additional data about its mechanism of action are needed prior to a full-scale randomized trial. RIM’s creator, Sandella (2016), describes the approach as one based on sudden transformation rather than gradual change, resulting from the opportunity to regenerate old and potentially un-integrated emotional memories in a context that promotes safety and feelings of competence instead of helplessness and vulnerability.

Different mental health treatments often share the same underlying mechanism of action, such as CBT and EMDR (the mechanism of exposure to stressors) or various antidepressant medications (the mechanism of increased serotonin availability). Verbal psychotherapeutic methods like RIM also may involve “common factors” that include a helping relationship, the chance to talk about problems, feelings of relaxation, and the expectation of positive results, among others. Treatments with similar underlying mechanisms often share features such as a typical level of efficacy, a common dose–response effect, or a similar side-effect profile; RIM’s atypical results therefore could imply a different mechanism of action (Hayes et al., 2007). Added to this argument is the fact that RIM’s originator *describes* the method as having a different mechanism of action, based on a primary skill of “neural witnessing” or following clients’ inner experiences without directing them, until the client arrives at a root-cause memory. This is different from EMDR in that the client does not construct a hierarchy of stressful situations, and indeed may have no conscious awareness of the root-cause memory prior to RIM. The facilitator then gently guides the client to safely speak or move within the traumatic memory, bringing a sense of empowerment and resolution into it. This approach is clinically more client-directed and body-centric than either CBT or EMDR.

### 1.2 Two Minds Theory

Two Minds Theory (TMT) is a neurocognitive model designed to explain the gap between people’s intentions and their behaviors based on the interactions of two independent brain systems (Cook et al., 2018a). These two systems, which operate in parallel, are the verbal and linear Narrative Mind and the non-conscious and multitasking Intuitive Mind. Although both mental systems are active continuously, only the Intuitive Mind operates fast enough to control behavior. TMT suggests that people’s immediate experiences and Intuitive-Mind reactions therefore have a greater effect on their behaviors. People’s beliefs and ideas, which are products of the Narrative Mind, occur secondary to experience and cannot directly affect behavior in the moment. The ongoing conscious experience of a person’s thoughts thus is more like a sports commentator than an executive manager, providing a constant narrative about thoughts and behaviors but not actually initiating them.

Two minds theory is built on neurocognitive research that shows a strong relationship between Narrative-Mind thinking and activity in the frontal lobes of the brain. The Intuitive Mind is more diffuse and may be thought of as a set of systems rather than a single brain area; it is associated with the brain’s default mode network of activation in the temporal and parietal lobes, and with deep-brain structures such as the limbic system (emotion), hippocampus (memory), thalamus (attention), basal ganglia (action selection), and nucleus accumbens (motivation). Together, the structures of the Intuitive Mind rapidly evaluate incoming stimuli and initiate behavioral responses without stopping to consult the consciously aware Narrative Mind (see Cook et al., 2018a for a review). TMT is an elaboration of dual-process models of mental activity that were popularized by Nobel laureate Dr. Daniel Kahneman (2011) and are now supported by extensive evidence (Evans, 2019; Hagger, 2016).

Two minds theory was developed separately from RIM, but may explain its effects. Trauma creates memories that are isolated from a person’s other experiences and sense of self, which then produce actions experienced as involuntary, such as high arousal or avoidance after a reminder of the trauma. In extreme cases, trauma can result in memories or mental states that are fully dissociated from a person’s conscious awareness. These findings are consistent with TMT, which suggests that all behavior has some level of disconnection from people’s conscious intentions. The more traumatic or stressful the person’s experience, the less integrated their memories will be, and the more likely the person is to have symptoms and problematic behaviors. The RIM approach to “regenerating” those memories (Sandella, 2016) therefore could reduce symptoms by making changes directly at the level of the Intuitive Mind. RIM is designed to tap into Intuitive-Mind brain functions, and then integrate these with the Narrative-Mind descriptions that tend to dominate people’s traumatic or unpleasant memories (Levine and Pizarro, 2004). From a TMT perspective, the reported beneficial effects of RIM are hypothesized to result from engaging the Intuitive Mind, particularly during the middle *see* phase of the procedure that involves accessing memories, and then from working through those experiences in the final *do* phase, which involves renewed engagement of the Narrative Mind. Once a memory has been re-experienced in a more positive way, the Intuitive Mind is able to generate the new, healthier response to similar future situations. Furthermore, these healthier responses should be more ego-syntonic because they also have been processed by the Narrative Mind. Cook et al. (2018a) argued that concerted action by the two mental systems is a particularly effective approach to increasing healthy behaviors.

### 1.3 Purpose of the current study

This study tested a specific TMT-based mechanism of action for RIM that involves participants shifting from a Narrative-Mind to an Intuitive-Mind mode of thinking over the course of a RIM session, and then back again. If our theory is correct, these changes should be detectable at the physiological level by examining brain activity patterns. Our hypotheses for each phase of RIM were derived from prior work on the differences in brain activity when people are experiencing more conscious (Narrative Mind or central executive network) versus more automatic and habitual (Intuitive Mind or default mode network) types of thinking (Brust-Carmona et al., 2017). To detect changes in activity for different areas of the brain across the three stages of RIM, we used a 5-lead electroencephalogram (EEG) device. EEG monitoring is a well-established technique that shows brain activity in particular areas (e.g., frontal versus temporal lobes). There have been no prior studies of RIM using brain monitoring or other biobehavioral metrics, which therefore was the purpose of the current study. Secondarily, we tested pre-post differences in nursing students’ mental health in order to replicate prior findings about the general effectiveness of RIM. As this study progressed, we also became aware of emerging literature on psychedelic medications as treatments for traumatic stress, and we added a questionnaire from the psychedelic research literature to determine whether participants’ subjective experiences during RIM were similar to or different from those reported by patients in psychedelic treatment studies.

## 2 Materials and methods

### 2.1 Participants

Participants were *N* = 30 undergraduate nursing students at the University of Colorado College of Nursing, recruited from a mental health course between February 2022 and April 2024. This study was reviewed and approved by the Colorado Multiple Institutional Review Board (protocol # 21-4215), and students provided written informed consent. Students were introduced to the study by their mental health simulation instructor (Ms. Aagaard) during a regular class session, who described RIM as a “verbal psychotherapeutic method to help people transform negative memories and feelings into more positive ones.” Students were provided with principal investigator (PI) Dr. Cook’s contact information. Ms. Aagaard did not know who followed up, and no other investigators were direct instructors with grading authority over these students. Interested students then contacted Dr. Cook and received an individual email or telephone call with more information including the study consent form. RIM might be contraindicated for people who are at elevated risk for psychosis; therefore, at the time of our initial meeting with students, we screened out anyone with bipolar disorder, psychosis, dementia, other cognitive disorders that interfere with reality testing, or with a recent history of hallucinogenic drug use. These complications were not expected in a population of generally high-functioning undergraduate nursing students, and no potential participants were excluded based on these criteria. During the course of the RIM session, one participant did report a history of 2 past (non-recent) experiences with psychedelic drugs.

**Figure 1:**
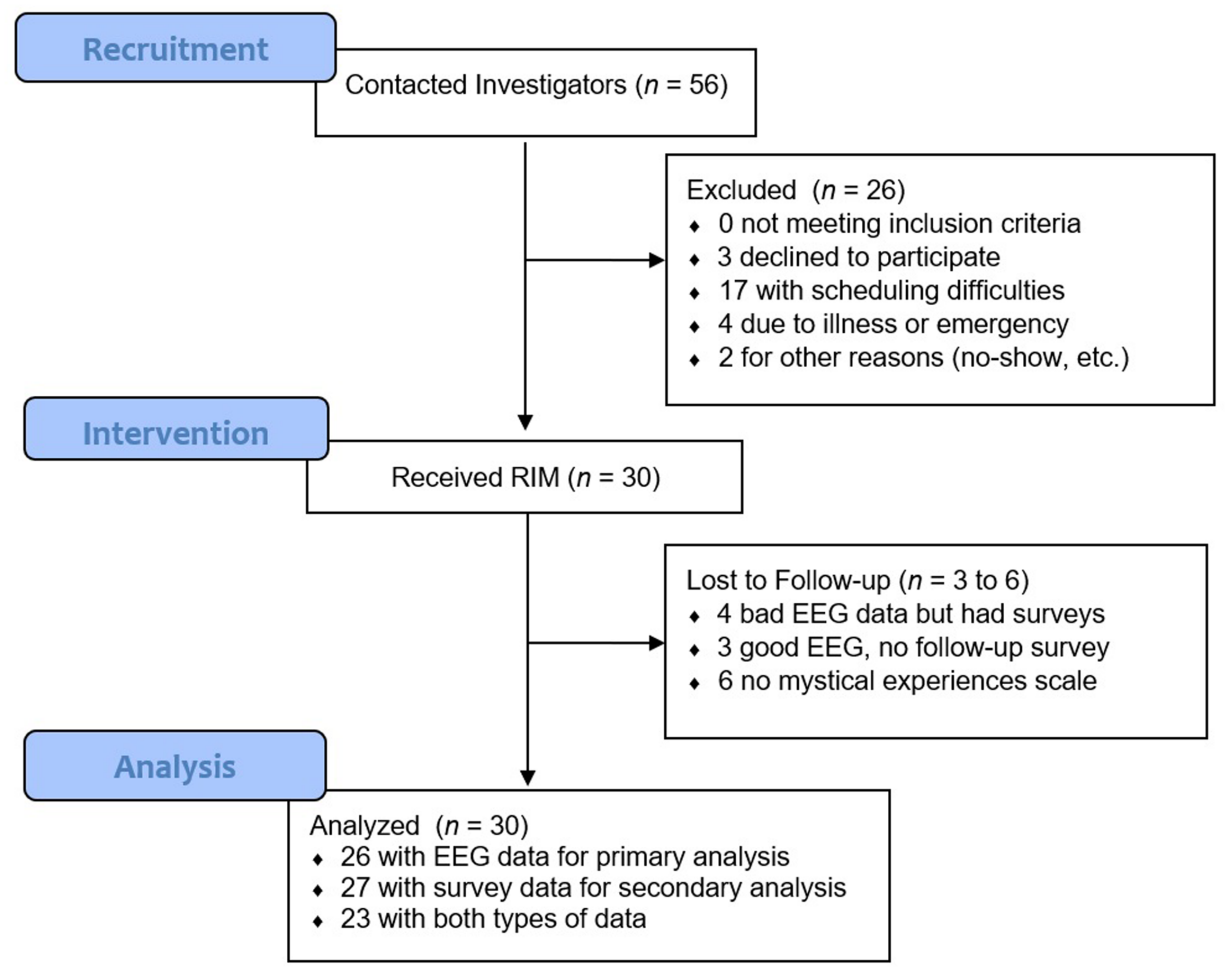
Study flow diagram showing recruitment and attrition. The most common reason for non-participation (17/23 cases) was scheduling difficulty. Other reasons for non-participation were illness (3 cases), personal emergency (1 case), miscommunication about the scheduled session time (1 case), and no-show for a scheduled study session (1 case).

Of 56 students who initially expressed interest in the study, 30 ultimately enrolled, a recruitment rate of 53.5%. Figure 1 shows study flow and students’ reasons for non-participation, which were mostly related to scheduling difficulties. Table 1 gives demographic characteristics of the 30 students who completed the RIM procedure. Of note, 27% of participants reported clinically significant levels of childhood adverse events (ACEs) based on the widely-used Kaiser ACEs scale (Felitti et al., 1998), even though this study did not specifically recruit participants based on childhood trauma. Additionally, 11 participants (37%) were receiving psychotherapy and 9 participants (31%) were taking psychotropic medication (primarily antidepressants) at the time they enrolled. This high level of mental health concerns among participants might simply reflect the overall level of vulnerability of nursing students, or might have been a characteristic of students who self-referred for a psychotherapeutic intervention study. There was no difference in gender between students who ultimately participated in the study and those who did not, *χ^2^* = 0.60, *p* = 0.44; we did not have race/ethnicity or age data for nonparticipants. There was no change in the recruitment rate over time, with an average enrollment around 55% during each of the 6 semesters that the study was recruiting. The only noted difference between participants and nonparticipants was that a significantly higher percentage of students who ultimately received RIM mentioned a specific mental health concern in their initial email (8/30), compared to those who ultimately did not participate (1/26), χ^2^ = 4.60, *p* = 0.03.

**Table 1:** Participant demographic characteristics.

Variable	*N* (%) or *M* (*SD*)
Gender	1 man (3.3%) 1 transman (3.3%) 28 women (93.3%)
Race and Ethnicity	1 Black, Hispanic (3.3%) 2 White, Hispanic (6.7%) 22 White, not Hispanic (73.3%) 1 Asian, Hispanic (3.3%) 4 Asian, not Hispanic (13.3%)
Age	29.8 (6.82)
Marital Status	21 single (70.0%) 5 married (16.7%) 2 living with partner (6.7%) 1 divorced (3.3%) 1 widowed (3.3%)
Years of Education	16.3 (1.48)
Employment Type	1 working full-time (3.3%) 18 working part-time (60.0%) 11 not working (36.7%)
Adverse Childhood Events score	3.70 (4.13)
≥4 Adverse Events	8 (27%)

Four or more adverse childhood events is the standard clinical cut-off level on the Kaiser ACEs scale, a level above which people are more likely to experience important effects in terms of their physical or mental health, education, employment, and/or relationships.

### 2.2 Procedure

After giving informed consent, including consent to audio-record, participants were scheduled for an individual, 1- to 2-h RIM facilitation session with Dr. Sandella, who is the original developer of this method. RIM facilitators are trained to follow the client’s lead, and to trust from experience that the client will end in a safe and empowered state of mind that is anchored in their body. Given that RIM is client-generated, and can sometimes draw out emotionally deep unconscious memories, it is important for RIM facilitators not to rush the client, and to create conditions of safety by going at the client’s pace. Every RIM session completes one piece of work from beginning to end, and the varying session length depended on the client’s experiences and needs. There was no comparison group in this exploratory, descriptive, mechanistic study, which was not designed as a test of RIM’s efficacy. All sessions were conducted in-person at Dr. Cook’s academic office, to facilitate real-time collection of EEG data. After asking the participant about their initial concerns or reason for participating, Dr. Sandella facilitated the RIM session. Standard RIM techniques were used to guide the participant through re-processing the spontaneous memories that arose during the session, as described in Sandella (2016). Dr. Cook was present as an observer, took notes, and functioned as a technician to monitor the EEG apparatus.

Participants wore a MUSE S (Gen 1) headband, which was connected to the investigators’ iPad using Mind Monitor EEG software (J. Clutterbuck.^1^) All EEG data were uploaded to a secure server for storage in Microsoft Excel format, and later analyzed using SPSS 29 (IBM Corp., Armonk NY) and Flourish data visualization software (Canva UK Operations Ltd., London.^2^) Brainwave data were monitored continuously throughout the RIM session, and recorded in sync with session audio. Dr. Cook’s process notes were later used to identify timing of the 3 RIM phases for each participant, and the audio recording was consulted as needed; Drs. Cook and Sandella reviewed the first 5 sessions together with 100% agreement about timing, based on the distinctive characteristics of the three phases as described in the Introduction. All determinations about the timing of RIM phases were made prior to analysis of brainwave data, to avoid biasing results.

Participants completed pre- and post-session questionnaires about their current mood, anxiety, motivation, stress, and self-esteem. Post-session only, participants completed an additional open-ended questionnaire about their experiences with RIM, any subjective changes in mental functioning, and any insights gained. This survey included qualitative items used in a prior RIM study (Boxwell and Eichler, 2006). Finally, an exploratory questionnaire asking about mystical experiences during the RIM session was added to the study about halfway through recruitment; some participants therefore completed the survey retrospectively and others completed it immediately at the end of the RIM session. As described below, this procedural variation did not lead to any systematic differences in survey results. At the end of the procedure, participants were paid $40 for their time and returned the EEG headband. Dr. Sandella followed up with each participant by telephone 1 week after their RIM session, and completed an additional brief telephone interview. In three cases participants asked for additional information, or that notes from their RIM session be shared with their regular psychotherapist; Dr. Cook handled these communications as requested. All participants were given the investigators’ contact information in case of any new questions, concerns, or mental health symptoms after the end of the study.

### 2.3 Apparatus

The MUSE S headband is an inexpensive, consumer-grade 5-lead EEG device used by psychotherapists or for personal meditation (InteraXon Inc.: Toronto, ON). Brainwave frequencies were measured by 4 forehead sensors placed above the left temporal (TP9), left frontal (AF7), right frontal (AF8), and right temporal (TP10) areas of the brain, as well as a right auxiliary sensor (AUX). MUSE devices have shown acceptable retest reliability as well as good agreement with two medical-grade EEG devices (Krigolson et al., 2017; Ratti et al., 2017), although the MUSE headband’s EEG readings have greater variability than the medical-grade device due to head movement or eye blinks. MUSE headbands have demonstrated concurrent validity based on an ability to successfully detect known task-related changes in brain functioning (Krigolson et al., 2017), predictive validity based on EEG changes observed during a learning task (Kovacevic et al., 2015), and discriminant validity based on detectable post-treatment differences between meditation training and math training (Bhayee et al., 2016).

### 2.4 Measures

#### 2.4.1 Demographics

Our demographic questionnaire included age, gender identity, current employment (part-time, full-time, not working), marital status, years of education, and race/ethnicity [select all that apply]. Participants were also asked about any current mental health treatment or medication, and completed the Kaiser ACEs scale on past traumatic experiences (Felitti et al., 1998).

#### 2.4.2 EEG amplitude by frequency, location, and time

Standard brainwave types are defined by frequency ranges in Hertz (Hz), as follows: delta (< 4 Hz), theta (4–8 Hz), alpha (8–13 Hz), beta (13–32 Hz), or gamma (32–100 Hz). Within these ranges, the amplitude of an EEG recording indicates the amount of that type of activity in a particular brain area at a specific point in time. The 5 frequency ranges can be analyzed as absolute values or percentages of total brain activity, which can also vary over time and by sensor location. Specific EEG frequencies have been shown to correlate with particular types of mental experiences (Kirmizi-Alsan et al., 2006). For instance, EEG activity in the delta range reflects slow, continuous, and non-conscious mental activity, particularly when delta waves predominate in the prefrontal cortex. Alpha- and beta-wave activity are both associated with conscious and effortful attention, with beta waves suggesting nervous alertness or active processing and alpha waves being more indicative of calm attention. Theta-wave activity is reliably associated with less-conscious mental states like drowsiness or meditation, and has been shown to correlate with unconscious impulses – e.g., when theta-wave activity is high, people report having to work harder to avoid making unwanted responses. Gamma waves, particularly in the frontal lobes, are a potential marker for meditative states (Braboszcz et al., 2017) or sudden insights (Benjaboonyazit, 2016). Total EEG activity in particular brain regions also has been shown to correlate with the shift from conscious, effortful thought (Narrative Mind, based on greater activity in the frontal lobes) to more automatic and habitual thought (Intuitive Mind, based on greater activity in the temporal lobes: Brust-Carmona et al., 2017). EEG cannot measure brain activity at the same level of resolution as PET or fMRI imaging, but it can effectively differentiate conscious awareness from less-conscious states (Huang et al., 2020; Rohaut and Naccache, 2017).

#### 2.4.3 Depressive symptoms

The Center for Epidemiological Studies Depression scale (CES-D: Radloff, 1977) is a widely used and well-validated survey that measures symptoms of depression. The measure consists of 20 items rated on a scale from 0 = *None of the Time* to 3 = *All of the Time*. The CES-D has shown strong internal consistency reliability (α = 0.86) as well as concurrent and discriminant validity versus a diagnostic interview in research on college students’ depression (Shean and Baldwin, 2008).

#### 2.4.4 Anxiety

The Generalized Anxiety Disorder scale (GAD-7: Spitzer et al., 2006) measures symptoms of anxiety with high reliability (α = 0.91). Items are measured on a 4-point frequency scale from 1 = *Not at All* to 4 = *Nearly All the Time*. The GAD-7 has strong construct validity based on factor analysis and concurrent validity based on correlations with other mental health symptom measures. High GAD-7 scores are also predictive of an anxiety disorder diagnosis based on a structured clinical interview, and on other measures of disability and medical utilization (Spitzer et al., 2006).

#### 2.4.5 Stress

An 8-item scale was previously created in an independent study by researchers at the University of Colorado College of Nursing, to measure nursing students’ experiences of stress and coping during the COVID-19 pandemic (Peterson et al., 2023). It includes items such as “I feel a sense of hope” and “I can manage my worries.” The stress scale was specifically designed to assess students’ ease or discomfort in the nursing student role, and therefore provided a different view of symptoms than the GAD-7 and CES-D. In the original study using this instrument, items were selected by mental health faculty based on their clinical expertise and their observation of nursing students’ challenges during the pandemic; the items’ construct validity was based on 100% expert consensus. Each item was rated on a 5-point scale from 1 = *Strongly Disagree* to 5 = *Strongly Agree*. In the previous study, the items on this scale had an internal consistency reliability of *α* = 0.84 (Peterson et al., 2023).

#### 2.4.6 Self-esteem

The Rosenberg Self-Esteem Scale (Rosenberg, 1965) is a widely used measure of self-esteem in college students. The scale consists of 10 items rated on a 4-point scale from 1 = *Strongly Disagree* to 4 = *Strongly Agree*, measuring either satisfaction with oneself (e.g., *I feel that I have a number of good qualities*) or dissatisfaction with oneself (e.g., *All in all, I am inclined to feel that I am a failure*). Half of the items measure dissatisfaction, and are reverse-scored to produce a single scale where high scores indicate satisfaction. This measure has satisfactory retest reliability, *r* = 0.82, and internal consistency reliability, *a* = 0.88 (Fleming and Courtney, 1984), as well as convergent validity with other self-esteem measures in an academic context (Reynolds, 1988).

#### 2.4.7 Motivation

The Herzog Motivation Scale (Herzog and Blagg, 2007) consists of 7 items that measure a participant’s motivation for a specified behavior, in this case “managing stress in healthy ways.” The definition of “healthy ways” was left up to the participant. Six of the items are on a 4-point scale and the final item is on a 7-point scale, which is prorated during scoring so that the items can be weighted equally in calculating a total scale score. This scale has internal consistency reliability of α = 0.79 and has shown predictive validity for later behavior (Cook et al., 2018b).

#### 2.4.8 Post-session satisfaction

At the conclusion of the RIM session, participants were asked “how would you rate your overall satisfaction with the RIM session,” on a scale from 1 = *Poor* to 5 = *Excellent*.

#### 2.4.9 Mystical experiences

The Pahnke-Richards Mystical Experiences Scale has been used to assess unusual states of consciousness in the psychedelic treatment literature (Stocker et al., 2024). It was completed immediately after the RIM session by 12 participants, and 1–6 months later by 12 other participants. We assessed the impact of this methodological variation with *t*-tests comparing the two administration methods, and found no differences, so all participants’ scores were combined for analysis. To minimize potential response bias, the questionnaire was labeled “states of consciousness scale” instead of “mystical experiences.” The Pahnke-Richards scale measures 7 different constructs, 5 of which were included in our version of the questionnaire for the current study: (1) changes in sense of self, (2) transcendence of time and space, (3) sense that the experience cannot be put into words, (4) sense of meaning, and (5) strong positive emotion. The Pahnke-Richards scale has a stable factor structure and concurrent validity with a measure of spiritual experience (Barrett et al., 2015). The constructs measuring inability to put an experience into words (“ineffability”) and a sense of experiences being more real than everyday life (“noetic quality”) have each been identified as particularly strong predictors of reduced depression in studies of psychedelics (Ko et al., 2022).

### 2.5 Statistical analysis

#### 2.5.1 EEG data processing

MindMonitor software provides real-time readouts of each brainwave frequency (delta, theta, alpha, beta, and gamma) at each of 4 primary sensor locations. Continuous EEG measurements were recorded in 1-s intervals by the MindMonitor software, resulting in a total of 71,809 observations from 30 participants. All EEG data were cleaned prior to analysis by examining the data for normality and Winsorizing extreme values to the 95^th^ percentile. Absolute numbers were used in tests of percentages to avoid negative values; for all other tests, raw numbers were used.

#### 2.5.2 Hypothesis testing

Self-report demographic data and scores on the Mystical Experiences Scale were analyzed descriptively. Changes on self-report surveys were tested by comparing the participants’ scores before versus immediately after RIM using paired *t*-tests. For the EEG data, we hypothesized that the 3 stages of RIM (*dip*, *see*, and *do*) would be characterized by different levels of brain activity between the frontal and temporal lobes, which are theoretically linked to conscious versus non-conscious mental activity. We also expected location-based differences in the amount of brainwave activity in specific frequency bands at each stage of RIM: For example, that theta-wave activity would increase in the temporal lobes from phase 1 (*dip*) to phase 2 (*see*), indicating a more meditative brain state. Data were analyzed using generalized linear models with time as a within-person variable, and RIM phases nested within persons to account for dependent observations.

#### 2.5.3 Power analysis

Our recruitment goal was *N* = 28, based on within-person tests comparing brain regions, 80% power at *α* = 0.05, and an expected effect size of *d* = 0.6 (based on Huang et al., 2020). In the actual analyses we corrected for inflated type 1 error using a Bonferroni correction, resulting in a final target significance level of *a* = 0.01 for all tests.

## 3 Results

### 3.1 Overall satisfaction with RIM

Participants reported very high overall satisfaction with RIM, *M* = 4.63 out of 5, *SD* = 0.57. Of 27 participants with post-session data, 18 gave the highest possible rating of “excellent” (67%), 8 said their experience was “very good” (30%), and only 1 gave a mediocre rating of “good” (4%), with no participants selecting the “fair” or “poor” categories. This is particularly noteworthy given our treatment-experienced sample, who were generally informed consumers of mental health services.

### 3.2 Pre-post improvement with RIM

Table 2 shows pre-post changes and effect sizes for each of the 5 survey measures, although we caution readers that these pre-post results do not demonstrate causation or treatment efficacy in the same way as a randomized controlled trial. Participants reported significantly lower levels of depression and anxiety, higher motivation for self-care, and lower stress after a single session of RIM, based on dependent-group *t*-tests with Bonferroni corrections for multiple comparisons. There was no change on the self-esteem scale. For mental health and motivation, effects were larger than the typical results of well-established psychotherapies such as CBT (Kolovos et al., 2016), although pre-post results are not directly comparable to effect size estimates from randomized trials. As in other RIM studies, benefits were seen in a single session, which is notably faster than the median 10–20 sessions needed to achieve therapeutic effects in traditional talk therapy (Lambert and Ogles, 2004). Effects were stronger than those seen in prior uncontrolled studies of RIM, which were in the *d* = 0.85–2.16 range (Cook, in press). This might be due to high intervention quality in the current study, where all RIM sessions were provided by the method’s original developer.

**Table 2:** Pre-post change in mental health indicators after 1 session of RIM.

Scale (abbreviation and range)	Pre-treatment *M* (*SD*)	Post-treatment *M* (*SD*)	Pre-post test and Cohen’s *d* effect size
Depression (CES-D: 0–60)	22.2 (5.37)	17.9 (5.07)	*t*(26) = 4.91, *p* < 0.001, *d* = 1.93
Motivation (Herzog scale: 1–4)	3.42 (0.43)	3.77 (0.26)	*t*(27) = 6.29, *p* < 0.001, *d* = 2.42
Mental Health (TOE scale: 1–5)	3.67 (0.57)	4.57 (0.39)	*t*(26) = 7.01, *p* < 0.001, *d* = 2.75
Self-Esteem (Rosenberg scale: 0–30)	16.7 (2.25)	16.7 (2.31)	*t*(27) = 0.00, *p* = 0.999, *d* = 0.00
Anxiety (GAD-7: 0–21)	8.07 (4.22)	3.54 (2.73)	*t*(27) = 6.79, *p* < 0.001, *d* = 2.61

For all analyses, *N* = 28 participants with both pre- and post-RIM self-report data. Three participants were not able to complete post-session questionnaires due to scheduling conflicts at the time of their RIM session and did not return the paperwork later.

### 3.3 Mystical experiences during RIM

**Figure 2:**
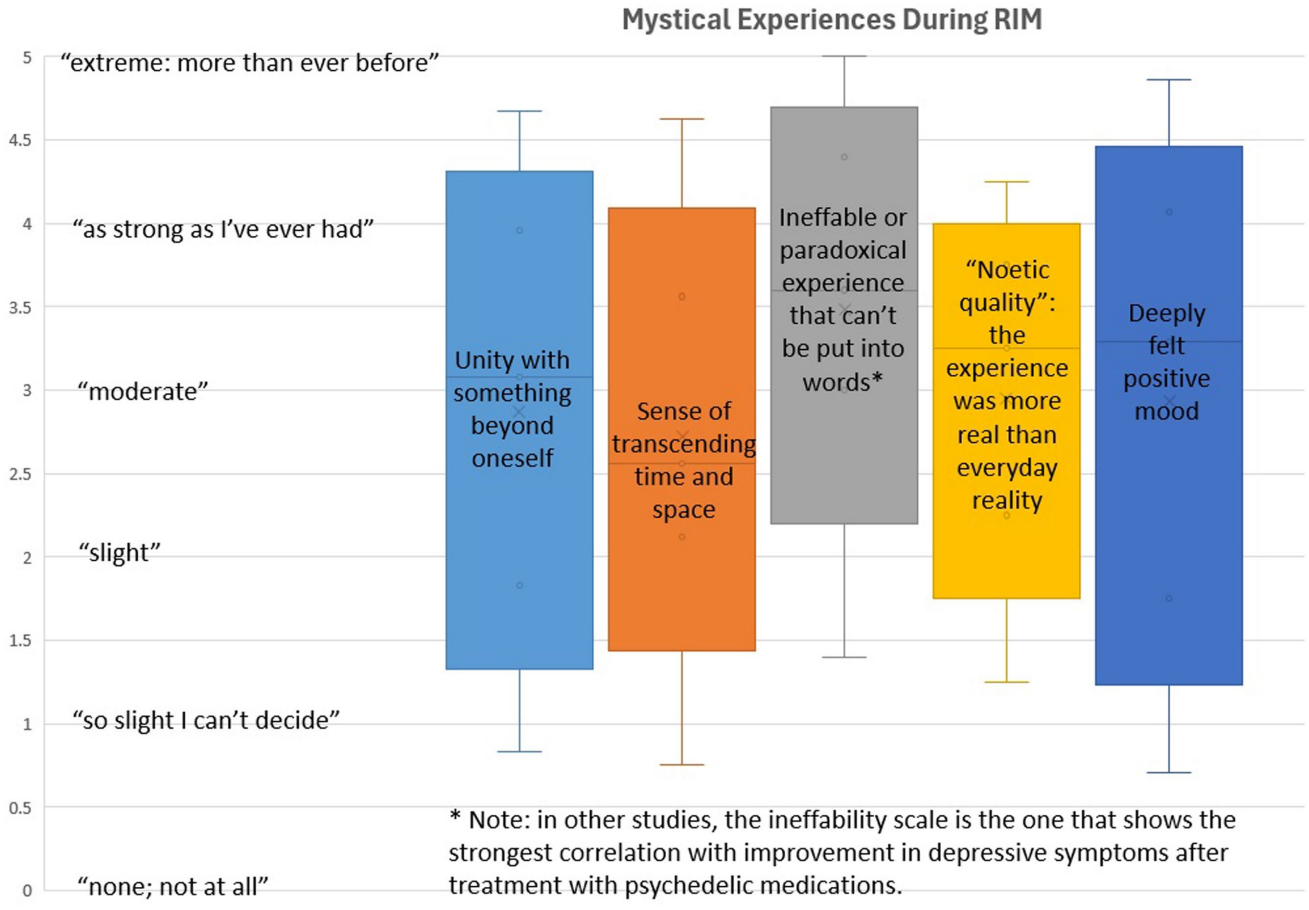
This figure shows the range of participants’ scores on the Mystical Experiences Scale, with elevated levels that are comparable to those seen in studies of psilocybin or ayahuasca. Furthermore, the strongest effects of RIM were seen in the specific subscales that have been most strongly linked to therapeutic improvement in those studies, which tested a purely pharmacological mechanism and did not include any direct therapist interaction during the psychedelic experience.

Participants reported very strong levels of mystical experience based on each of the 5 subscales of the Pahnke-Richards scale, as shown in Figure 2. These are again exploratory, descriptive results that suggest possible mechanisms of action for RIM, rather than proof that RIM causes mystical experiences. It is noteworthy that on each of the subscales, at least 25% of participants (as indicated by the upper hinge of the box) reported that their experience during the RIM session was “as strong as I’ve ever had.” These levels of mystical experience are comparable to those commonly seen in studies of psychedelics (Ko et al., 2022), which is noteworthy because RIM does not involve the administration of any drug. It is also noteworthy that the single highest subscale, ineffability, measures a dimension of mystical experience that has been found to predict symptom improvement in some prior studies of psychedelics (Ko et al., 2022). As an aside, one RIM participant who reported 2 prior experiences with psychedelics said that her RIM experience was in some ways stronger than either of those medication-induced psychedelic states, and that it involved a greater sense of control throughout the process.

### 3.4 EEG changes during RIM

**Figure 3:**
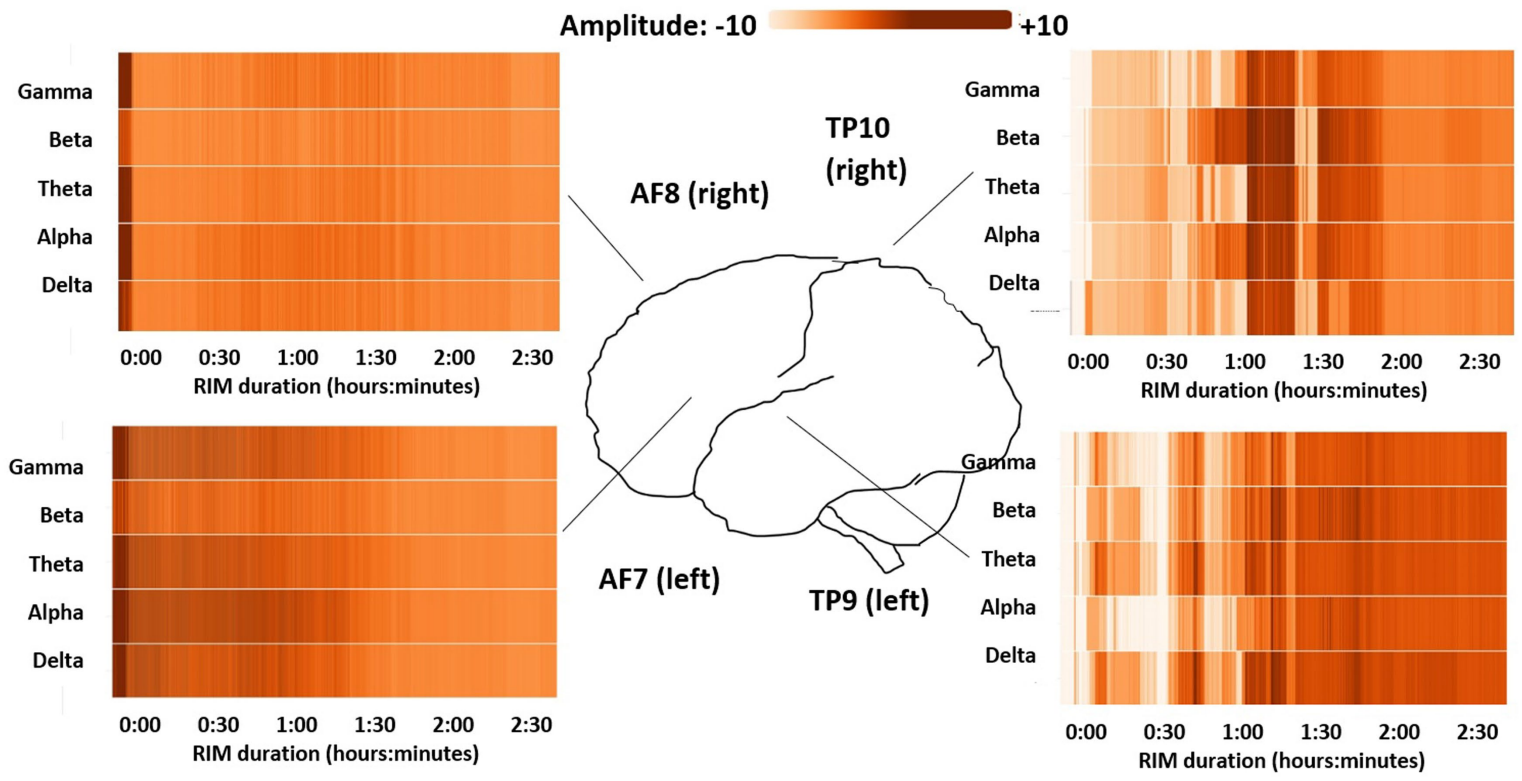
Heat maps showing changing brain activity levels over the course of the RIM session, by EEG frequency range (*y* axis) and time (*x* axis). Darker shades indicate more intense activity in that bandwidth, while lighter colors indicate less activity of that type. Progressively lightening bars for the AF7 and AF8 sensors show decreasing frontal-lobe (Narrative Mind) activity, while darkening bars for the TP9 and TP10 sensors show increasing temporal-lobe (Intuitive Mind) activity as the RIM session progressed.

The most notable characteristic of the EEG readings was a strong increase in temporal-lobe activity across the duration of the entire RIM session, with a smaller concurrent decrease in frontal-lobe activity (Figure 3). This effect was seen equally in all 5 EEG frequency bands. The net effect was that more than half of all measured brain activity shifted to the temporal lobes, which are theoretically more connected to the Intuitive Mind according to TMT. The temporal-lobe percentage of total brain activity was significantly different across each of the 3 RIM phases based on generalized linear models with RIM phases nested within persons, Wald χ^2^ = 11.0 × 10^4^, *p* < 0.001. This shift to the temporal lobes was particularly pronounced during the middle *see* phase of RIM, which is also when participants described the strongest imagery (Cook, in press).

At the same time that the frontal lobes were becoming less active, a higher percentage of frontal-lobe activity shifted into the slow-wave delta and theta frequencies. We failed to confirm *a priori* hypotheses about the percentage of activity in specific bandwidths. However, when we compared total percentages for the 3 highest EEG bands (alpha, beta, and gamma) to the lowest 2 EEG bands (delta and theta), we found a significant fast-frequency decrease in the frontal lobes from the initial *dip* to the middle *see* stage of RIM, Wald χ^2^ = 32.9 × 10^4^, *p* < 0.001, along with a corresponding fast-frequency increase in the temporal lobes, Wald χ^*2*^ = 116.7 × 10^4^, *p* < 0.001. During the final *do* stage of RIM, the temporal-lobe percentages of fast-frequency EEG activity continued to increase while the frontal-lobe percentages moved back toward baseline. These findings suggest that in the frontal-lobe areas most clearly connected to conscious thoughts and intentions, more activity during the *see* stage mirrored sleep or meditation, while active mental processing moved to the temporal lobes. During the final *do* stage, some faster-frequency activity returned to the frontal lobes, but this continued to increase in the temporal lobes as well, which might suggest greater integration across brain areas.

**Figure 4:**
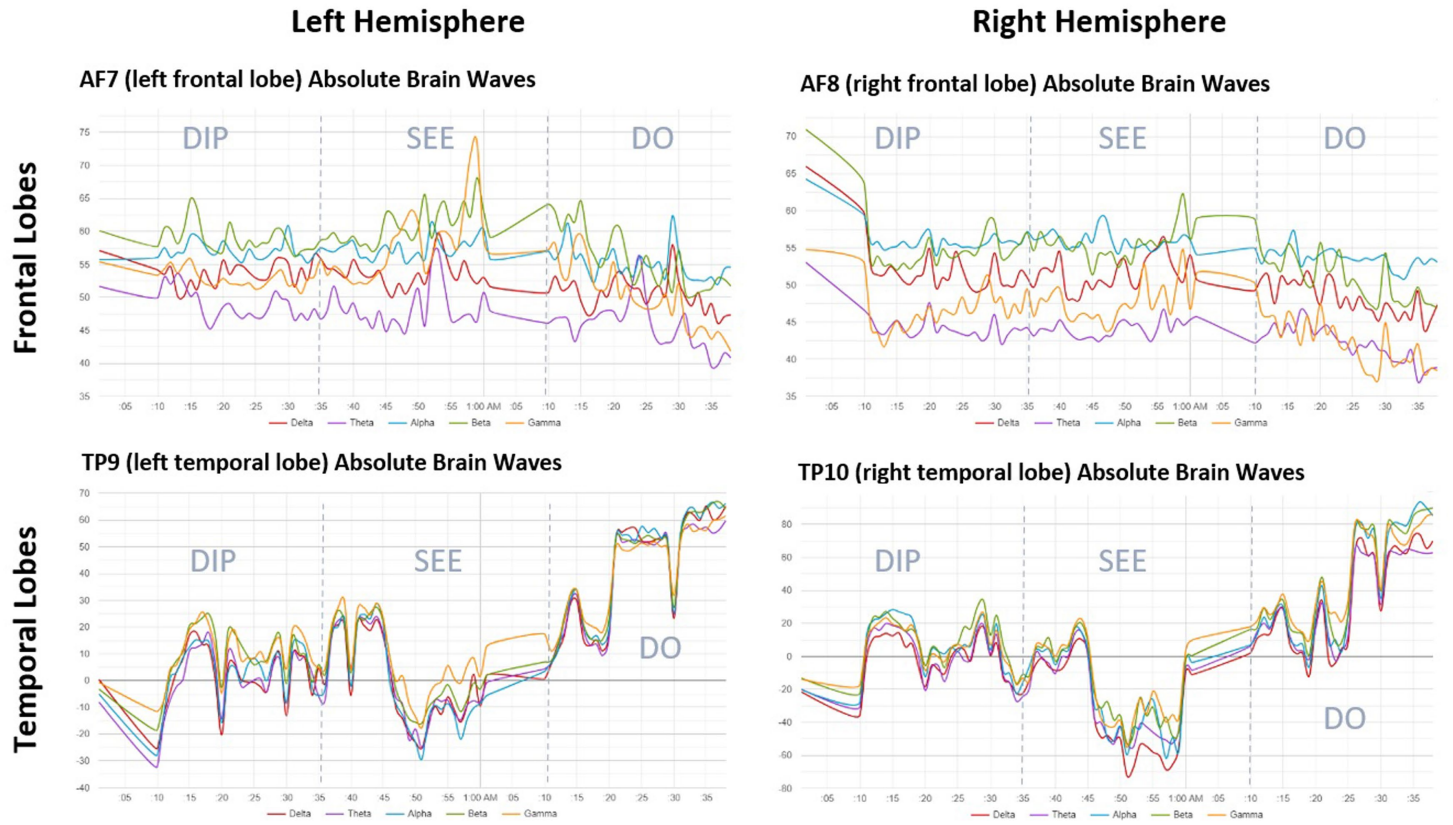
This figure shows absolute brainwave frequencies by EEG sensor over the duration of the RIM session, averaged across participants (*N* = 30), and illustrates different patterns of activity in the frontal vs. the temporal lobes. Note the lower absolute amplitude (scaling from 35 to 75) in brainwave readings from frontal lobes, compared to the higher amplitude (scaling from −80 to 100) the readings from the temporal lobes. The difference in y-axis scaling means that there were greater overall levels of brain activity in the temporal lobes, consistent with activation of the Intuitive Mind. Similar to Figure 3, temporal-lobe activity increases as time proceeds from left to right, while frontal-lobe activity remains steady or decreases slightly. The interesting spike in beta- and gamma-wave activity around the 1-h mark in all sensors coincides with the portion of the RIM session where participants were most often engaged in intense imagery and inner dialogue. There were few left–right differences even during an imagery-intensive procedure, suggesting no differential hemispheric activation during RIM.

As a final step, we examined the pattern of specific EEG frequencies in each brain area over time. Figure 4 is a composite illustration created by averaging readings across all 30 participants, and standardizing the duration for each phase of RIM. As in Figure 3, this image shows relatively consistent brain activation across EEG frequency bands, and increasing temporal-lobe activity over time, but different patterns of activity in the frontal- versus temporal-lobe sensors. Note that the *y*-axis scales are not identical for the frontal- versus temporal-lobe sensors, as the frontal-lobe activity occurred within a narrower range. Figure 4 also shows some interesting activity in individual EEG bandwidths that might be relevant for future research, such as a temporary increase in gamma-wave activity toward the end of the middle *see* phase of RIM. This is a point in the procedure where participants often report the most intense and personally meaningful imagery (Cook, in press), and gamma EEG readings are sometimes thought to correlate with creative thinking or insight (Benjaboonyazit, 2016). For the most part, however, changes over time tended to be simultaneous across all EEG bandwidths, and far outweighed the differences between individual EEG frequency bands at any given point in time.

## 4 Discussion

RIM is a novel, imagery-based, body-centered psychotherapeutic approach that is hypothesized to help people achieve rapid remission of mental health symptoms by allowing them to regenerate troubling memories in a new and healthy way. Our investigation found support for rapid symptom improvement, consistent with prior nonrandomized and pilot studies of RIM (Boxwell and Eichler, 2006; Elle and Alberts, 2023; Ellis Ecke, 2018; Kneier, 2016; McDonald, 2017; Prah, 2015; Waidtl, 2015). Effect sizes were larger than in prior RIM research, perhaps because all interventions were delivered by RIM’s original developer. It is a well-established finding in the psychotherapy literature that treatments have their strongest effects in the hands of the masters who developed them (Hill and Lambert, 2004). Participants also reported extremely high levels of satisfaction with RIM, with two-thirds of participants selecting the highest category on a 5-point scale. These very positive results were seen after a single 1–2 h session of RIM, which is a faster time to response than the most common medication or psychotherapy approaches for stress and trauma (Lambert and Ogles, 2004; Warden et al., 2007).

This study also had two important findings about RIM’s mechanism of action. First, descriptive results showed that participants reported mystical experiences during their RIM session. Although these results are merely exploratory and hypothesis-generating, participants’ reported experiences were of a type and intensity that is most often seen in studies of psychedelic medications. Furthermore, the strongest results were reported on an ineffability subscale that indicates difficulty putting an experience into words, a dimension of mystical experience that predicts symptom improvement in some studies of psychedelics for refractory depression (Ko et al., 2022). The phenomenon of ineffability also may relate to the posited transition from a Narrative-Mind to an Intuitive-Mind mode of thinking during RIM, because TMT identifies language with the Narrative Mind (Cook et al., 2018a), so that Intuitive-Mind experiences are by definition more difficult to put into words. Similarly, some psychedelic studies show stronger integration across brain areas after treatment (Daws et al., 2022), which mirrors the finding of greater synchronization between the frontal and temporal lobes during the final phase of RIM.

Second, tests for differences in EEG readings across RIM phases confirmed that observable changes in brain activity occurred over the course of a RIM session. Our original hypotheses were based on differences in the relative amount of brain activity in specific EEG frequencies for certain brain locations and points in time. These very precise hypotheses were not confirmed, perhaps because any changes in the percentage of activity at specific frequencies were relatively subtle. Instead, we observed a dramatic increase in overall temporal-lobe activity, which continued throughout the RIM session and involved more fast-cycle brainwaves, while frontal-lobe activity decreased and showed more slow-wave delta and theta EEG activity. The shift into sleep- and meditation-related frequency bands in the frontal lobes matches participants’ reported sensation of feeling “sleepy” or “out of it” during RIM. The concurrent increased fast-wave activity in the temporal lobes is consistent with the theorized TMT-based mechanism of action for RIM, which involves Intuitive-Mind re-experiencing of memories. This contrast was particularly noticeable during the most imagery-intense part of RIM, the middle *see* phase. The observed decrease in frontal-lobe activity, particularly in fast-wave EEG bands, parallels recent findings about “hypofrontality” (i.e., decreased frontal-lobe activity) during exercise, which may account for aerobic exercise’s mental-health benefits (Dietrich, 2006). Like the findings about RIM recipients’ subjective mystical experiences, this study’s EEG results again parallel psychedelic studies’ findings that show decreased frontal-lobe activity during imagery-laden mystical experiences (Carhart-Harris et al., 2017).

Taken together, our findings fit well with TMT’s proposition that behavior change originates in the Intuitive Mind. EEG results present a consistent picture of temporal-lobe processing during the RIM procedure, which is associated with the Intuitive Mind under TMT, and appears clinically as nonlinear and spontaneously experienced rather than consciously controlled mental activity. This is followed by increased frontal-lobe activity, which is associated with the Narrative Mind under TMT, and appears clinically as a re-integration in which experience comes into conscious awareness so that it is available to both mental systems for future reflection and guidance of behavior. Parallel processing by participants’ two mental systems may help to explain RIM’s rapid reduction of mental health symptoms. We posit that similar mechanisms may in fact be at work in psychedelic treatments or other rapid-relief therapies that are currently under investigation.

TMT is of course not the only potential explanation for the observed findings: Other theories that emphasize nonconscious and body-oriented emotional processing such as psychodynamic theory (Schottenbauer et al., 2008) or polyvagal theory (Porges, 2016) might also be used to interpret the observed EEG results. One theory that does *not* fit well with the observed results, however, is the dominant cognitive model of psychotherapy, which implies that frontal-lobe executive control over interpretations and behaviors is the key to effective symptom management (Zalta, 2015).

### 4.1 Limitations

First, the current study did not include a randomized control group. This means we cannot be certain about RIM’s efficacy for symptom reduction, which was not the goal of this mechanistic study. In particular, we note that pre-post effect sizes are often larger than the effects seen when comparing an active treatment to a no-treatment or usual-care control group, and that without a psychotherapeutic comparison group one cannot rule out the possible effects of common factors. To draw causal inferences about RIM’s effects, this clinical approach needs to be more extensively evaluated in large-scale randomized trials. Second, as noted above, the current study may show “ideal” rather than “typical” effects of RIM because the intervention was delivered with high fidelity by its original developer (Dr. Sandella); the facilitator’s expectation of positive results also might explain some of the apparent benefits. The potential for expectancy bias in results was partly mitigated by the fact that an independent investigator (Dr. Cook, who has no direct affiliation with RIM) performed all analyses. Third, the mystical experiences scale’s introduction partway into the study resulted in methodological variability, with some surveys completed immediately and others months after the RIM session. We tested for differences between participants who completed the questionnaire immediately versus later, with no evidence that this created a problem for interpretation, but the change in methods still might have biased our results in unknown ways. Fourth, this study involved a nonclinical sample of nursing students, although this concern is mitigated to some extent by the high levels of anxiety, depression, and trauma reported by participants, and by the fact that about one-third were also receiving mental health treatment. Nevertheless, this study’s small sample size and recruitment of students from a single school limits its generalizability. Finally, although we observed meaningful EEG differences across the three phases of RIM, they were not the differences we originally expected. Some of that was likely our own naïveté when formulating hypotheses at this early stage of RIM research, and the patterns that we did observe in the data seem unmistakable. However, our findings are still ultimately *post hoc*, and therefore replication is essential. In light of these limitations, our findings should be viewed as suggesting an effect and a potential mechanism of action that must be confirmed in a subsequent randomized trial with more rigorous statistical controls.

### 4.2 Directions for research and practice

With preliminary evidence of RIM’s efficacy in treating symptoms of traumatic stress, and the existence of a large unmet need for fast-acting and highly efficacious mental health treatments, the most important next study should be a randomized controlled trial of RIM. Such a study should screen participants to ensure that they meet formal diagnostic criteria for one or more mental health conditions (e.g., PTSD), and should randomly assign participants to RIM or a comparison group. Appropriate comparisons might include usual care (e.g., antidepressant medication prescribed by a non-specialist), or CBT (a standard of care treatment that works by a very different mechanism focused on the Narrative Mind). Including an alternative form of psychotherapy as a comparison group in the next RIM study would help to establish its effects above and beyond those based on common factors found in all forms of talk therapy. Given the overlap between participants’ experiences during RIM and those reported in psychedelic studies, it would also be interesting to study RIM versus psychedelic medication in a comparative effectiveness trial. Further mechanistic studies of RIM are also warranted, both to confirm the EEG results obtained here and to use more sophisticated brain-imaging techniques. One barrier to studying RIM with PET or fMRI methods is that RIM requires ongoing verbal communication between the interventionist and participant, something that would be difficult to achieve while the participant is inside a loud and movement-constricting fMRI machine. However, the increase in temporal-lobe EEG amplitude seen here during both the *see* and *do* phases might point to a lasting change in brain activity that could be measured after the conclusion of the RIM session. Further EEG research could also follow up on exploratory findings such as an apparent increase in gamma-wave activity toward the end of the *see* phase, to determine whether this type of brain response was indeed correlated with significant insights based on participants’ retrospective reports or qualitative analysis of RIM session transcripts. Qualitative methods also could be used to explore the RIM proposition that sharing power within the session results in clients uncovering inherent resources within themselves that are currently being obscured or overridden by the Narrative Mind.

RIM is currently available in community settings, delivered by certified facilitators who are trained by the RIM Institute (Denver, CO). However, there are relatively few trained RIM practitioners compared to other psychotherapeutic methods. If RIM’s effects are confirmed in a randomized trial, then broader dissemination would be warranted. In the meantime, RIM remains a promising practice that could be considered by mental health professionals or others who work with patients seeking rapid symptom relief or who have not been helped by currently available forms of treatment. EEG monitoring might be a useful addition to research on RIM training; it could, for example, be used as a criterion to determine whether new RIM facilitators are successfully engaging a patient’s Intuitive Mind.

## 5 Conclusion

New methods like RIM challenge assumptions about the primacy of the logical, reasoning Narrative Mind as the seat of decision-making and behavior, and suggest a new way of engaging people’s inherent resources that may have been previously unavailable or actively suppressed by conscious thinking. TMT provides a neurocognitive model that suggests an important role of the Intuitive Mind in everyday behavior, which could help to explain the benefits reported by people who receive RIM. If RIM is indeed able to activate the Intuitive Mind directly, followed by re-integration of new experiences with the Narrative Mind, then it can potentially affect symptoms in ways that traditional talk therapies operating mainly at the Narrative-Mind level do not. RIM appears to have a novel mechanism of action, which may involve some of the same types of subjective mystical experiences as psychedelic medications currently under investigation. Better understanding of the mechanisms of action behind RIM could provide useful data for training RIM practitioners in how to use the method successfully, and can contribute to our understanding of how psychotherapy or other mental health treatment works in general. The current findings also can support efforts to design new and more effective interventions that capitalize on the brain’s underlying structure and function, in order to help people make faster and more extensive changes in their behavior.

## Data Availability

The de-identified raw data supporting the conclusions of this article will be made available to qualified investigators upon request.

## Footnotes

^1^ https://mind-monitor.com/

^2^ https://flourish.studio
